# Consumer acceptability of interventions to reduce *Campylobacter* in the poultry food chain

**DOI:** 10.1016/j.foodcont.2013.06.005

**Published:** 2014-01

**Authors:** L.A. MacRitchie, C.J. Hunter, N.J.C. Strachan

**Affiliations:** a Institute of Biological and Environmental Sciences, School of Biological Sciences, University of Aberdeen, Cruickshank building, St Machar Drive, Aberdeen, AB24 3UU, Scotland; b School of Geography & Geosciences, University of St Andrews, Irvine Building, North Street, St Andrews, KY16 9AL, Scotland

**Keywords:** *Campylobacter*, Chickens, Interventions, Food poisoning, Consumer, Acceptability

## Abstract

Reducing human *Campylobacter* cases has become a priority for the UK Government. However the public's views on acceptability of interventions to reduce *Campylobacter* in poultry production are poorly understood in the UK and in other countries around the world. The objective of the study was to investigate how increasing awareness and knowledge changes consumer acceptability of interventions that reduce human campylobacteriosis in the poultry food chain. This approach is readily applicable to other risks and associated interventions. It involved a survey of the views of consumers in the Grampian region in North East Scotland. This found that better hygiene practices on farm, freezing chicken meat and vaccination of chickens were acceptable to the majority of participants (95%, 53% & 52% respectively) whilst irradiation and chemical wash of chicken meat were acceptable to <50%. Increasing consumer awareness by providing information on the *Campylobacter* disease burden in humans increased the number of participants finding them acceptable. However, chemical wash and irradiation remained the least acceptable interventions, although highly effective at reducing *Campylobacter*, and were found to be never acceptable to >50% of respondents. It was found on average that food poisoning concern, previous awareness of *Campylobacter* and living in rural or urban areas had either no or little effect effect on the acceptability of interventions. Further, previous awareness of *Campylobacter* did not influence consumer concern of harmful bacteria on chicken meat. Overall, findings indicate that increasing consumer acceptability of the most effective interventions is likely to be a difficult process.

## Highlights

• Better farm hygiene is the most acceptable intervention to reduce *Campylobacter*.
• Chemical wash and irradiation were found to be the least acceptable interventions.
• Heightened consumer awareness of disease increased intervention acceptability.
• Previous awareness and concern of *Campylobacter* did not influence acceptability.
• Increasing acceptability of interventions is likely to be a difficult process.

## 1 Introduction

### 1.1 The *Campylobacter* problem

*Campylobacter* is the most common cause of bacterial gastrointestinal disease in the developed world (Gabriel et al., 2010) and caused a reported 70,298 cases in the UK in 2010 (Defra, 2011). However, most cases are underreported and the actual number in the UK is estimated to be over 500,000 per year (Tam et al., 2012). Symptoms of human campylobacteriosis include diarrhoea, abdominal pain and nausea, which tend to last for 5–7 days with minor relapses occurring in 15–25% of cases (Blaser & Engberg, 2008). 10% of cases are hospitalised (Bessell et al., 2010) and 0.2% end in death (Adak, Meakins, Yip, Lopman & O'Brien, 2005). Post infection complications associated with campylobacteriosis include Guillian–Barré syndrome, reactive arthritis and inflammatory bowel disease (Moore et al., 2005). In addition, the financial burden of *Campylobacter* was estimated to be £583 million in 2008 in the UK (Food Standards Agency, 2010).

Many pathways of *Campylobacter* infection have been identified, but the consumption of contaminated poultry is considered to be the most common source of campylobacteriosis in humans (Moore et al., 2005). The association with chicken was demonstrated when chicken products were removed from sale due to dioxin contamination in Belgium and in a 40% reduction in human *Campylobacter* cases (Vellinga & Van Loock, 2002). Therefore a decrease of *Campylobacter* on chicken meat is crucial for reducing the number of human infection cases.

*Campylobacter* infection in poultry begins at the farm caused by, for example, poor biosecurity, contaminated feed or transmission from one crop to the next. Therefore good hygiene and biosecurity practices are required to be in place to avoid contamination of flocks (Gibbens, Pascoe, Evans, Davies & Sayers, 2001). The infection is asymptomatic and once in a flock *Campylobacter* is rapidly transmitted by the faecal-oral route throughout the broilers (Wassenaar, 2011). The bacteria can then survive during poultry processing (stages comprise of transport, slaughter, processing and preparation) through to human consumption (Hartnett, Kelly, Gettinby & Wooldridge, 2002) and causing subsequent illness. However at each of the process stages interventions either are in place or can be implemented to control *Campylobacter*.

Biosecurity practices at the farm include disinfecting poultry houses, boot dips (Galanis, 2007), fly screens (Hald, Sommer & Skovgård, 2007) disinfecting equipment and vehicles, and treating the flock water supply (Wassenaar, 2011). Alternative practices to antibiotic additives in broiler feed currently being investigated are probiotics (Gaggìa, Mattarelli & Biavati, 2010), bacteriocins (Svetoch & Stern, 2010), bacteriophage (Monk, Rees, Barrow, Hagens & Harper, 2010) and vaccination (De Zoete, van Putten & Wagenaar, 2007). Interventions at the slaughter stage include steaming, forced air chill, electrolysed oxidising water as a disinfectant agent (Wassenaar, 2011), chemical wash (Keener, Bashor, Curtis, Sheldon & Kathariou, 2004), crust freezing (Rosenquist et al., 2009) and irradiation (Havelaar et al., 2007). In the home, good hygiene is important to avoid cross contamination and chicken meat should be cooked properly to prevent consumption of potentially harmful food.

### 1.2 Consumer acceptability of interventions

In general, the acceptability of interventions by consumers is a potentially important determinant for government decision making as effective policy initiatives are reinforced by public preferences and concerns (Cope et al., 2010). Consumers may be more willing to accept new interventions where they have a role in choosing these themselves rather than having them imposed by government and industry (Krebs, 2001).

Factors that may influence acceptability of interventions include: the level of concern that people associate with interventions (e.g. irradiation intervention may be perceived to be risky in itself); the awareness the public has about the intervention; the willingness to voluntarily accept it (Breakwell, 2007); and, the severity or extent of the consequences the consumer would have to endure if it was not in place (e.g. a higher incidence of *Campylobacter* cases) (Renn, 2008). Previous research indicates that concern of meat being safe to eat more generally and an awareness of *Campylobacter* and are factors that can influence acceptability of interventions (Breakwell, 2000,Fife-Schaw & Rowe, 1996). Other factors that may play a role in influencing acceptability of interventions are cultural perception (Renn, 2005), individual attitudes, demographic characteristics (Breakwell, 2000) such as where people live, and measures that the public put in place to protect themselves (Renn, 2008); e.g. a change in shopping habits to avoid unsafe products (Dillaway, Messer, Bernard & Kaiser, 2011).

It is suspected that those living in rural areas could have greater awareness because they have higher incidence of campylobacteriosis compared to urban residents in Grampian (Strachan et al., 2009).

Investigation into acceptability of interventions to improve food safety has been carried out for other meats, for example, a European study on acceptability of interventions and new technologies in the beef (De Barcellos et al., 2010; Van Wezemael, Verbeke, Kügler & Scholderer, 2011), turkey meat (Yan, Lee, Nam, Min & Ahn, 2006) and pork (Mørkbak, Christensen & Gyrd-Hansen, 2012) food chain.

Recent publications from New Zealand and the UK have highlighted consumer views on interventions to reduce *Campylobacter* on poultry meat (Gilbert & Cressey, 2008; Jordon & Stockley, 2010). In 2008 the New Zealand Food Safety Authority published a report investigating (by telephone questionnaire) consumer knowledge, attitudes and beliefs with respect to *Campylobacter* and poultry. Chemical wash was found to be the least favoured intervention, with the most popular intervention being stricter farm management. A quarter of respondents were found to be willing to pay a 10–20% premium on safe chicken through stricter farm management (Gilbert & Cressey, 2008). However, the New Zealand study did not investigate the factors that could influence acceptability of interventions.

In 2009 the Food Standards Agency (FSA) used consumer discussion forums to determine the levels of awareness and understanding of *Campylobacter* and collate opinions for reducing levels of foodborne disease (Jordon & Stockley, 2010). The process included showing participants a film of the poultry process and *Campylobacter* risks. The study found that consumers recognised that *Campylobacter* posed a risk to public health, and that ‘on farm’ interventions, lactic acid spray, heat treatments and packing interventions to prevent cross-contamination were most favoured. Other factors found to influence consumer attitudes on interventions were convenience, additional cost, the effect on consumer experience, food safety and ethical concerns. However, it should be noted that this research used qualitative methods through focus groups.

Although there has been some research conducted on the acceptability of interventions for *Campylobacter* in poultry (Gilbert & Cressey, 2008; Jordon & Stockley, 2010) there is a gap in understanding with regards to how changing consumer awareness and knowledge on both the burden of disease and efficacy of interventions might lead to changes of opinion on intervention acceptability (Breakwell, 2000). This paper develops an approach to address this which is applicable to other risks and their associated interventions.

### 1.3 Aim and objectives

The aim of the research reported here, therefore, was to investigate consumer acceptability of a range of interventions in the poultry food chain. Specific objectives were to:

I) determine the most and least acceptable interventions;
II) ascertain the effect on the acceptability of interventions by providing additional information (i.e. increasing awareness) on *Campylobacter* and by suggested effectiveness caused by the interventions on acceptability; and,
III) investigate if acceptability was influenced by prior concern and awareness of *Campylobacter* as well as living location (rural or urban).

The paper concludes by considering the implications of findings for developing strategies to reduce human campylobacteriosis.

## 2 Material and methods

### 2.1 Study population

Research was focused on the Grampian region of North East Scotland (*n* = 210) (population of 519,979). This region was selected as studies conducted in Grampian have identified consumption of chicken as a major source of campylobacteriosis (Strachan et al., 2009) and Grampian has a high incidence rate of infection (138.8/100,000 population in 2010) (Locking, Browning, Smith-Palmer & Brownlie, 2012). From the Grampian population a sample of residents living in rural (*n* = 103) and urban (*n* = 107) areas was collected, where rural was defined as postcode sectors with <200 people per km^2^ (Strachan et al., 2009). Given that half of the Grampian population reside in rural areas and half in urban areas (Dehring & Dunse, 2006), the sample population was collected to represent this. Also taken into consideration for the sample population was to stratify the male and female participants to represent the shopper profile which is 25% male (sample contained 26% male *n* = 56) (Gil, Tobari, Rose & Penn, 2009). The sample population was not stratified by occupation or age due to lack of shopper profile data these fields.

### 2.2 Study design

A survey was carried out using a paper-based questionnaire (see Supplementary data). Convenience sampling was used to obtain data from the targeted Grampian population between February and March 2011. Rural participants were sought from five towns in Aberdeenshire (Aboyne, Banchory, Ellon, Huntly and Inverurie), to obtain a spread of respondents from over Grampian. As the researcher wandered around these towns and came across people, they were asked if they would like to take part in the study. Urban participants were sought in Aberdeen city and included utilising the opportunity of a hand washing campaign in a shopping mall. The questionnaire was completed by the participant although the researcher was present to collect completed questionnaires. Face to face delivery was carried out (the researcher would read out the questionnaire and fill in the responses) for those who sought assistance. The socio-demographic characteristics of the data collected can be seen in Supplementary information. The study was approved by the School of Geosciences ethics committee at the University of Aberdeen.

The questionnaire included nine closed questions and four open questions. The closed questions were used to gather information on concern, acceptability, awareness and the affect of increasing awareness on acceptability. The open questions were used to collect information on the socio-demographics of the participants.

The following interventions to reduce *Campylobacter* in the poultry industry were selected from the literature and included in the study to assess acceptability:

• Vaccination of live chickens on the farm
• Feeding chickens additives that kill bacteria on the farm
• Better hygiene practices on the farm
• freezing chicken meat at processing plants
• Chemical/chlorine wash of chicken meat at processing plants
• Steaming of chicken meat at processing plants
• Irradiation treatment of chicken meat at processing plants

To find out about acceptability participants were asked if they found the interventions to be acceptable, unacceptable or don't know. To ascertain if increasing awareness of *Campylobacter* changed acceptability opinion, information on the burden of human campylobacteriosis in Grampian including number of cases, financial burden and symptoms was provided. Following was a question to rate acceptability by effectiveness of the treatments (a range of hypothetical values of effectiveness on a five point scale, 10%, 25%, 50%, 75% and 90% case reduction, were provided).

### 2.3 Data analysis

Categorical data from the questionnaire were analysed in PASW Statistics 18 software (IBM SPSS Statistics, USA). Excel was used to calculate the percentages and 95% binomial confidence intervals to express the participant response to; concern of raw chicken carrying harmful bacteria, awareness of *Campylobacter*, and acceptability of interventions to reduce *Campylobacter*. To determine if there were significant differences between observations (e.g. acceptability differences between rural and urban participants) Monte Carlo sampling (@RISK, Palisade Ivybridge, United Kingdom) of binomially distributed variables with corrections for multiple comparisons using the Bonferroni method (Salkind, 2007) was used. Age, postcode and gender were explored to investigate that the acceptability results were not inadvertently biased by participant demographics (see Supplementary data). This was achieved by plotting bar graphs and associated 95% binomial confidence intervals.

## 3 Results

### 3.1 Most and least acceptable interventions

**Fig. 1:**
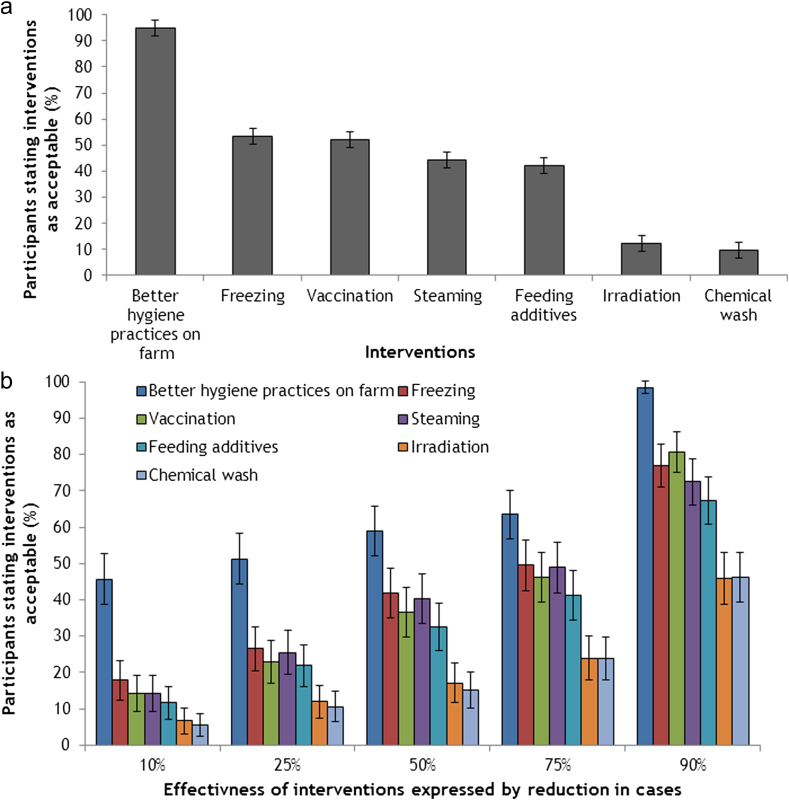
a) Percentage of participants (*n* = 210) who stated interventions as acceptable before additional information was provided (95% binominal CI). b) Percentage of participants (*n* = 210) who stated each intervention as acceptable (an accumulated percentage) on an effectiveness scale after additional information on *Campylobacter* infections had been provided (95% binominal CI).

The intervention that was found to be acceptable to most of the participants (prior to providing participants with information on the *Campylobacter* burden) was better on farm hygiene practices (95% acceptability), with the least acceptable chemical wash (10% acceptability) and irradiation (12% acceptability) (Fig. 1a). The four remaining treatments (steaming, vaccination, freezing and feeding additives) were found to be acceptable to around half (42%–53% acceptability) of the participants.

### 3.2 The effect of providing additional information on acceptability of interventions

**Fig. 2:**
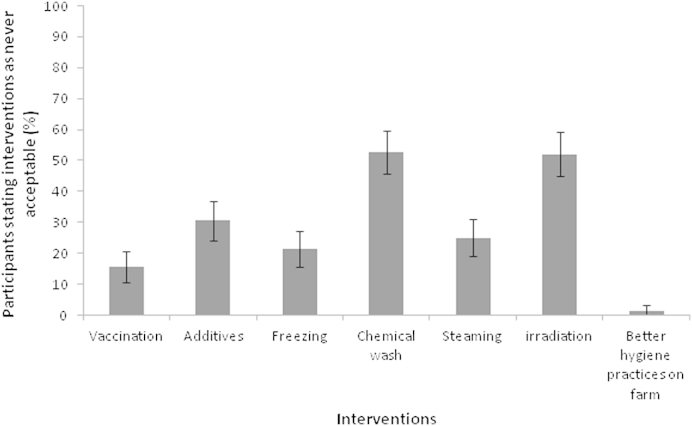
Percentage of respondents (*n* = 210) stating that an intervention is never acceptable even after participants were exposed to additional information on *Campylobacter* infection and the intervention was 90% efficient at reducing cases (95% binomial CI).

It was found that acceptability of interventions increased with greater effectiveness, which was measured by a hypothetical reduction in human *Campylobacter* cases (Fig. 1b). Better on farm hygiene practices always had the greatest number of respondents finding it acceptable (46%–99% acceptability when hypothetical effectiveness increased from 10% to 90%) compared to the other interventions (Fig. 1b). This was followed by freezing (18%–77% acceptability), steaming (14%–72% acceptability), vaccination (14%–81% acceptability), and feeding additives (11%–67% acceptability). Chemical wash and irradiation always had the fewest number of respondents indicating acceptability and no matter what the level of effectiveness they were never acceptable to 53% and 54% of participants (Fig. 2.).

### 3.3 Other factors affecting the acceptability of interventions

**Fig. 3:**
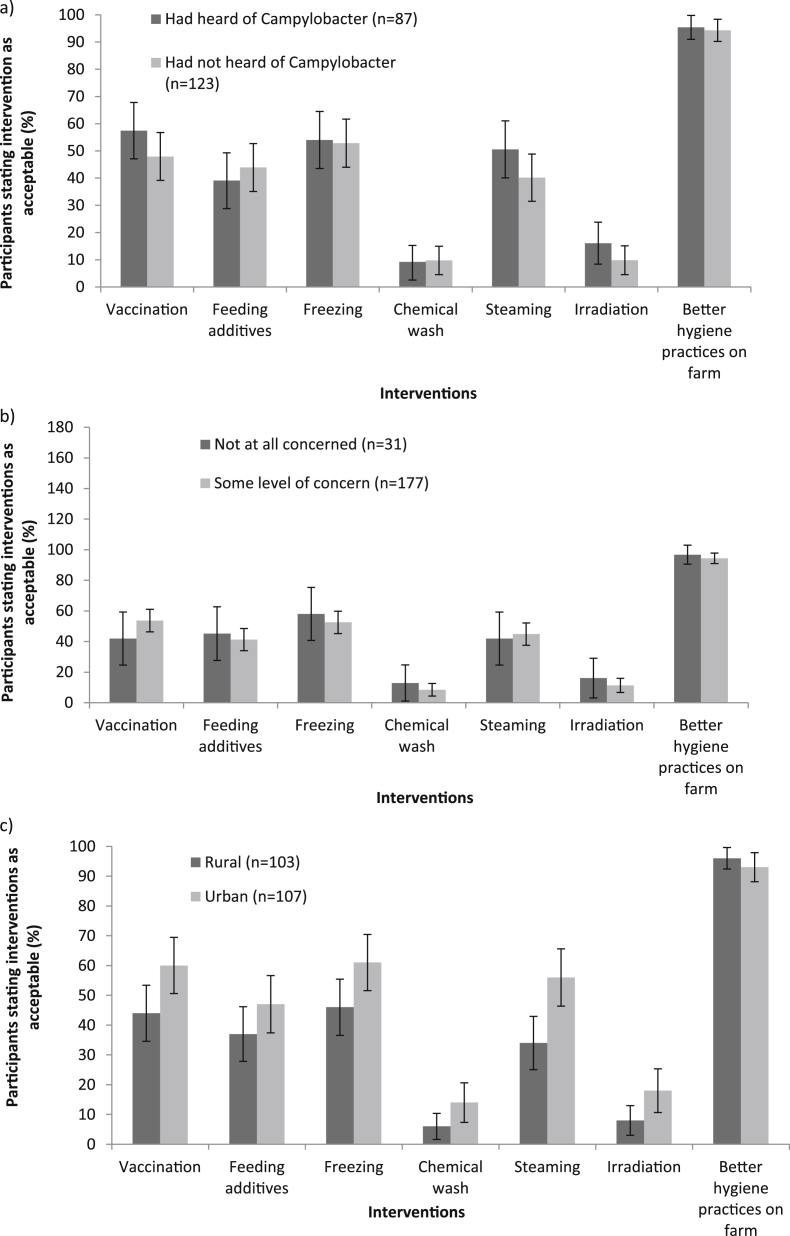
a) The effect of previous awareness of *Campylobacter* infection on intervention acceptability (95% binominal confidence intervals). b) Relationship between concern of harmful bacteria on food and acceptability of interventions (95% binomial CI). c) Percentage of rural and urban participants stating an intervention as acceptable (95% binomial CI).

It was found that 41% (95% CI 48.1–34.8) of participants had previously heard of *Campylobacter* but that of these only 48% associated it with chicken. Prior awareness of *Campylobacter* was not found to influence the proportion of participants stating an intervention as being acceptable (Fig. 3a). This was the case for all of the interventions studied.

Some 85% (95% CI 89.9–80.3) of participants were concerned about harmful bacteria on raw chicken meat and 28% (95% CI 34.0–21.8) of these were extremely concerned. There was no statistically significant relationship, however, between reported concern and acceptability of any intervention (Fig. 3b). The comparison of rural and urban residents showed there was no significant difference in the acceptability of interventions, except for steaming (*P* = 0.008) which was found to be more acceptable to urban residents. The proportion of respondents who had some level of concern towards harmful bacteria did not depend on prior awareness of *Campylobacter* and 94% of participants thought that a reduction in *Campylobacter* cases was needed (data not shown).

## 4 Discussion

This study investigated the acceptability of interventions to reduce *Campylobacter* contamination of chicken meat. The new findings presented in this paper are the acceptability of interventions in relation to *Campylobacter* and assessing the impact of certain factors which include concern of harmful bacteria, awareness of *Campylobacter* and living location on acceptability.

### 4.1 Most and least acceptable interventions

The current research found that a majority of respondents chose better on-farm hygiene practices as the most acceptable method. Chemical wash and irradiation were the least acceptable. Similar findings were observed in a New Zealand study (Gilbert & Cressey, 2008) in that stricter farm management was the most popular option whereas irradiation or chemically treating chicken came last. On-farm interventions were also viewed favourably by the FSA consumer focus groups (Jordon and Stockley, 2010). Therefore, it appears that consumers are not in favour of interventions applied directly on chicken meat. There was a distinct trend starting from on-farm hygiene at the top; then vaccination, freezing, feed additives and steaming grouped in middle ground and finally chemical wash and irradiation at the bottom of the scale.

### 4.2 The effect of increased awareness

After additional information was read by the participants to increase their awareness of *Campylobacter* by including incidence in Grampian, symptoms and intervention effectiveness, the trend of acceptability was not altered. However, increased awareness of the effectiveness of various interventions did make them more acceptable. For example, there was an average increase of 26% in acceptability after increased awareness of the presence of *Campylobacter* and its dangers to public health (based on 90% efficiency of interventions). To better understand the importance of these findings they need to be compared with the actual effectiveness of each intervention.

### 4.3 The effectiveness of interventions

The reported effectiveness of interventions included in this study is limited but the following is what the evidence suggests. There has been evidence reported that irradiation and chemical wash were effective in minimising *Campylobacter* on chicken meat. For example, irradiation can reduce *Campylobacter* by 20.8 log (cfu/carcass), (Havelaar et al., 2007) and chemical wash (e.g. application of chlorine in spin chillers at 35 ppm) has been a major tool in reducing human campylobacteriosis in New Zealand (New Zealand Food Safety Authority, 2009). Freezing was found to reduce microbial counts by 1–2 log (Rosenquist, Nielsen, Sommer, Nørrung & Christensen, 2003) and steaming by 1.8 log (Rosenquist et al., 2009). Feeding additives t broilers, for instance bacteriocins (a type of feed additive) were found to reduce *Campylobacter* to non-detectable levels (Connerton, Timms & Connerton, 2011; Svetoch & Stern, 2010). Investigation into vaccination of chickens provided evidence of inconsistent success in reducing *Campylobacter* (Hermans et al., 2011). Better on-farm hygiene practices have been reported to lower the risk of *Campylobacter* colonisation by 95% (Havelaar et al., 2007).

### 4.4 Weighing up the options

This study has highlighted a discrepancy between public acceptability and the reported effect of interventions, especially as regards to irradiation and chemical wash procedures. On the other hand, the high approval rate as well as effectiveness of the improved on-farm hygiene option makes it a viable intervention to implement. The only problem is that farmers may feel they are being targeted to take all of the responsibility; farms already have bio-security practices in place and making further improvements would add to their costs. Therefore, incentives such as subsidies may be required to encourage farmers to adopt additional bio-security measures (Fraser, Williams, Powell & Cook, 2010) or attracting financial bonuses for *Campylobacter-*negative flocks (Tustin et al., 2011).

### 4.5 The cost factor

Reports show that introducing new interventions would be beneficial in reducing the number of human campylobacteriosis cases. However, there would also be an associated increase in costs for producers, the production process and for consumers themselves. A possible alternative would be to introduce a premium price for *Campylobacter*-free chicken meat. This idea was investigated in the New Zealand study of consumer knowledge and attitudes (Gilbert & Cressey, 2008), where participants selected a 10–20% premium for safer chicken depending on the acceptability of the intervention. Introducing an offset premium cost for *Campylobacter*-free chicken meat could provide a solution and would be worth investigating in a future study among consumers.

### 4.6 Concern, awareness and living location

The results presented here demonstrate that the level of concern of harmful bacteria did not appear to have a bearing on a respondent's views regarding interventions. This is surprising because one would have expected that the greater the concern, the more acceptable the intervention. It was expected that the current study would have shown lower concern due to reported UK data which saw a decline in food poisoning fears in recent years. For example, when people were asked through FSA consumer attitude studies whether they were concerned about food safety issues, 71% were concerned in 2000 compared with 30% in 2011 (Food Standards Agency, 2007,Food Standards Agency, 2011). At the same time, New Zealand, reported that a large number of respondents were concerned about harmful bacteria on raw chicken meat – 85% and 92% respectively (Gilbert & Cressey, 2008).

It had been assumed that participants with previous awareness of risks posed by *Campylobacter* would find interventions more acceptable than those without. Nevertheless, this was found not to be so. The results show that the Grampian population was less aware of the risks (41%) than their counterparts in New Zealand at 70% (Gilbert and Cressey, August 2008) but more than those in other countries, like Ireland at 8% (Riordan, Cowan & McCarthy, 2002) and the US at 7% (Lin, Jensen & Yen, 2005).

Living location was suggested as a factor affecting acceptability of interventions but it was discovered to have no influence, except for steaming. It had been presumed that as *Campylobacter* incidence is greater in rural areas of Grampian (MacRitchie, Hunter & Strachan, 2012), this could have been reflected in rural dwellers finding interventions more acceptable. The study did find that there were no significant differences in awareness between rural and urban residents.

### 4.7 Consumer awareness strategies

It has been established that risk communication strategies have a role in combating food poisoning (Miles, Braxton & Frewer, 1999) and that it is beneficial for consumers to have access to information on disease burden and interventions in order to construct informed opinions. Therefore, risk communication can alter public acceptability of interventions to reduce *Campylobacter*. However, this study found that >50% of participants would never find irradiation or chemical wash acceptable. Even if a large awareness campaign was mounted it might not be cost effective or achieve the desired effect of changing views on the acceptability of these interventions.

The methodology applied here of providing information to consumers and observing whether attitudes change, is readily applicable to a number of other areas. It would be interesting to observe how favourable conditions could be created to achieve substantial changes in intervention acceptability in other areas of public health and safety.

Another strategy would be to implement the most effective interventions despite the views of consumers. This occurred in New Zealand when chemical wash on chicken meat was carried out (New Zealand Food Safety Authority, 2009) although this had not been favoured by the public (Gilbert & Cressey, 2008). However, consumers did benefit, as the incidence of human campylobacteriosis decreased (Sears et al., 2011). Whatever strategies are chosen, public health authorities should recognise that it is difficult to change consumer opinion (Fig. 1).

## 5 Conclusions

This study provides new data on public acceptability of interventions that could be used to reduce human campylobacteriosis cases employing a transferable approach. The results suggest that the most acceptable method was better hygiene practices on the farm and that chemical wash and irradiation were the least attractive. It is likely that the best way to control *Campylobacter* would be a combination of interventions throughout the ‘farm to fork’ process. Prior awareness of *Campylobacter* or concern about food poisoning were shown to have no effect on the acceptability of interventions. On the other hand, increasing awareness and providing additional information on the effectiveness of various interventions was found to be a notable determinant of consumer acceptability. However, both chemical wash and irradiation were never acceptable to >50% of the participants. The findings reported in this paper are important for both government and industry when developing strategies to decrease *Campylobacter* contamination on chicken meat and subsequently reduce food poisoning.
