# Supplementary material for: Consumer acceptability of interventions to reduce Campylobacter in the poultry food chain
Source: Food Control. 2014 Jan;35(1):260–6. doi: 10.1016/j.foodcont.2013.06.005 (PMC4029083; doi:10.1016/j.foodcont.2013.06.005)
Supplement: Supplementary file 1 [file mmc1.docx]

**Supplementary Information**

The study population consisted of Grampian residents who were questioned between February and March 2011. The inclusion criteria for the survey were for participants to be over the age of 17 and were resident in the Grampian region. The data collected included age, gender, occupation, postcode and if the participant had children of school age. The study population was approximately evenly divided between living in rural and urban areas.

Table A1 – Socio-demographic data of the survey sample population.

| **Socio-demographic** | | | |
| --- | --- | --- | --- |
|  |  | No. | % |
| Q9 | Female | 154 | 73 |
|  | Male | 56 | 27 |
| Q8 | 20-34 | 60 | 29 |
|  | 35-44 | 30 | 15 |
|  | 45-64 | 74 | 36 |
|  | 65+ | 42 | 20 |
| Q12 | Rural | 107 | 51 |
|  | Urban | 103 | 49 |
| Q11 | Employed | 109 | 52 |
|  | Unemployed | 2 | 1 |
|  | Retired | 54 | 26 |
|  | Higher education | 18 | 7 |
|  | Other | 27 | 13 |
| Q10 | Those with children | 73 | 35 |
|  | Those with no children | 137 | 65 |

To determine if there was a socio-demographic difference in the percentage of participants finding an intervention as acceptable they were grouped by gender and age. A higher percentage of males found irradiation acceptable compared to females (Fig. A1.). The age groups showed no significant difference for any of the interventions (Fig. A2.).


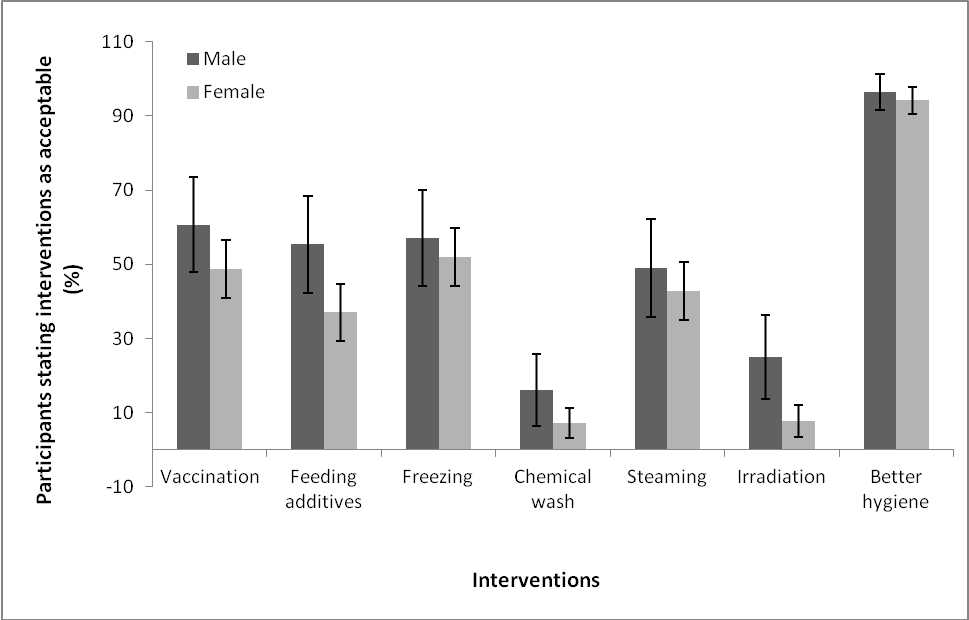


Fig. A1. – Percentage of male and female participants stating an intervention as acceptable (95% binominal CI’s)


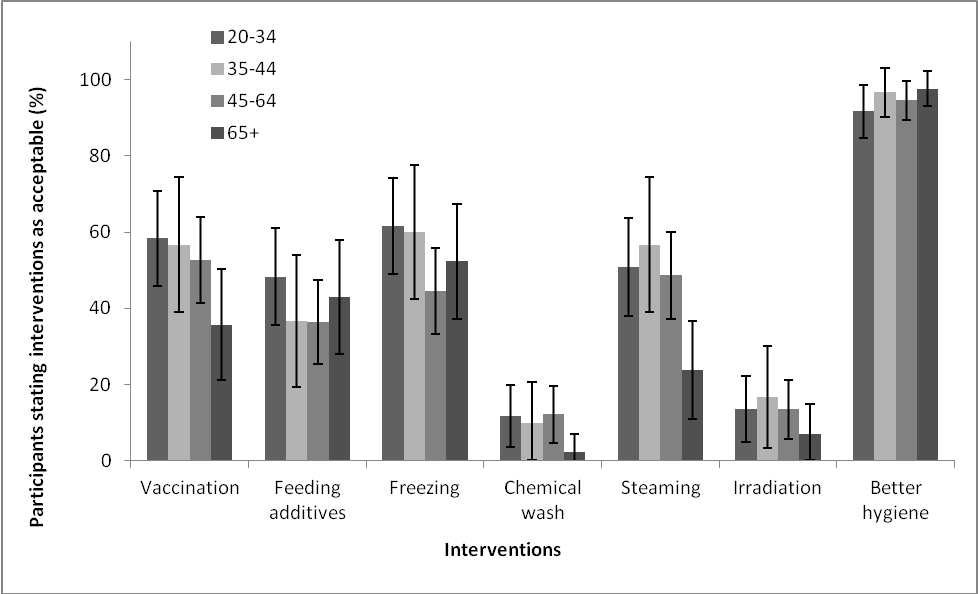


Fig. A2. – Percentage of participants (stratified by age group) stating an intervention as acceptable (95% binominal CI’s)

**Consumer Questionnaire**

Thank you for agreeing to take part in this research. This study is carried out by the University of Aberdeen and we are collecting views from the general public about their concern regarding germs on chicken and the acceptability of methods used to treat chicken. This questionnaire is anonymous. Please could you fill in answers, **circle and tick where appropriate**, the questionnaire should take 5-10 minutes.

1. Are you **concerned** that raw chicken may carry harmful bacteria that can cause food poisoning in people?

**Please circle** how concerned you are on a scale from 1 to 5.

1. Not at all concerned
2. Extremely concerned

There are a number of treatments that farmers and food businesses can use to reduce the level of harmful bacteria that cause food poisoning in people.

1. Which of the following treatments to reduce the risk of food poisoning caused by bacteria in chicken do you find **acceptable**?

**Please tick**.

| **Treatments** | **Acceptable** | **Unacceptable** | **Don’t Know** |
| --- | --- | --- | --- |
| Vaccination of live chickens at farm | 🞐 | 🞐 | 🞐 |
| Feeding chickens additives that kill bacteria at the farm | 🞐 | 🞐 | 🞐 |
| Freezing chicken meat at processing plants | 🞐 | 🞐 | 🞐 |
| Chemical/chlorine wash of the chicken meat at processing plants | 🞐 | 🞐 | 🞐 |
| Steaming the chicken meat at processing plant | 🞐 | 🞐 | 🞐 |
| Irradiation treatment of chicken meat at processing plant | 🞐 | 🞐 | 🞐 |
| Better Hygiene Practices on farm | 🞐 | 🞐 | 🞐 |

1. Have **you heard** of *Campylobacter*?

🞐 Yes 🞐 No

1. **If yes**, how did you hear about it?

|  | News |  | Radio |  | Outbreak |
| --- | --- | --- | --- | --- | --- |
|  | TV |  | Internet |  | Trade Journals |
|  | School education |  | Through work |  | Personal experience |
|  | Other ……………………………………………………………………………………………………………………………………. | | | | |

1. Do you associate chicken with *Campylobacter*?

🞐 Yes 🞐 No

The following is information on Campylobacter, please read it before completing the rest of the questionnaire. You do not need to change your previous answers, there are no right or wrong answers.

*Campylobacter* causes about **300,000 cases** of food poisoning each year in the **UK**.

Chicken has been found to be the main source of human illness caused by *Campylobacter*.

Each year *Campylobacter* on chicken causes:

- - about **150,000 cases**
  - about **15,000 hospitalisations**
  - **76 deaths** per year

**Grampian** region has the **4^th^ highest** **incidence** of Campylobacter cases in Scotland.

The approximate cost of *Campylobacter* illness in the UK is **£500 million** per year.

The infection causes the following symptoms:

- - diarrhoea
  - severe stomach cramps
  - fever
  - general discomfort

1. Do you think a reduction in Campylobacter cases is needed?

🞐 Yes 🞐 No (If no please go to question 8.)

1. What **reduction** in **cases** would need to be achieved by the introduction of each treatment to chicken before you would consider it **acceptable**?

**Please tick** the level of reduction you would find appropriate for the following treatments**:**

| **Treatments** | **About a 10% reduction in cases**  **(15,000 cases)** | **About a 25% reduction in cases**  **(37,500 cases)** | **About a 50% reduction in cases**  **(75,000 cases)** | **About a 75% reduction in cases**  **(112,500 cases)** | **About a 90% reduction in cases**  **(135,000 cases)** | **Never acceptable** |
| --- | --- | --- | --- | --- | --- | --- |
| Vaccination of live chickens at farm | 🞐 | 🞐 | 🞐 | 🞐 | 🞐 | 🞐 |
| Feeding chickens additives that kill bacteria at the farm | 🞐 | 🞐 | 🞐 | 🞐 | 🞐 | 🞐 |
| Freezing chicken meat at processing plants | 🞐 | 🞐 | 🞐 | 🞐 | 🞐 | 🞐 |
| Chemical/chlorine wash of the chicken meat at processing plants | 🞐 | 🞐 | 🞐 | 🞐 | 🞐 | 🞐 |
| Steaming the chicken meat at processing plant | 🞐 | 🞐 | 🞐 | 🞐 | 🞐 | 🞐 |
| Irradiation treatment of chicken meat at processing plant | 🞐 | 🞐 | 🞐 | 🞐 | 🞐 | 🞐 |
| Better Hygiene Practices on farm | 🞐 | 🞐 | 🞐 | 🞐 | 🞐 | 🞐 |

About You

1. Age ……………………………………………………………………
2. Gender Male 🞐 Female 🞐
3. Do you have children up to School age? 🞐 Yes 🞐 No
4. Occupation …………………………………………………………
5. Postcode ……………………………………………………………..
6. Is there anything you would like to add about *Campylobacter* or about specific intervention measures?
